# Supplementary material for: Effectiveness and Safety of Ayurvedic Medicines in Type 2 Diabetes Mellitus Management: A Systematic Review and Meta-Analysis
Source: Front Pharmacol. 2022 Jun 8;13:821810. doi: 10.3389/fphar.2022.821810 (PMC9213670; doi:10.3389/fphar.2022.821810)
Supplement: Supplementary file 1 [file DataSheet1.zip › Supplementary Material/Forest and Funnel Plots/Punica granatum/LDL-C.pdf]

Punica granatum - LDL-C

Study	Intervention N	Intervention Mean	Intervention SD	Comparator N	Comparator Mean	Comparator SD	Mean Diff. with 95% CI	Weight (%)
Faghihimani 2017	37	114.00	34.00	37	101.00	21.00	13.00 [0.12, 25.88]	38.60
Khajebishak 2019a, Khajebishak 2019b	26	93.00	24.30	26	90.60	34.10	2.40 [-13.69, 18.49]	34.86
Grabež 2020	19	123.34	43.69	18	144.61	27.45	-21.27 [-44.93, 2.40]	26.54
**Overall**							0.21 [-18.09, 18.51]	

Heterogeneity: $\tau^2 = 182.62$, $I^2 = 71.10\%$, $H^2 = 3.46$

Test of $\theta_i = \theta_j$: Q(2) = 6.29, p = 0.04

Test of $\theta = 0$: z = 0.02, p = 0.98

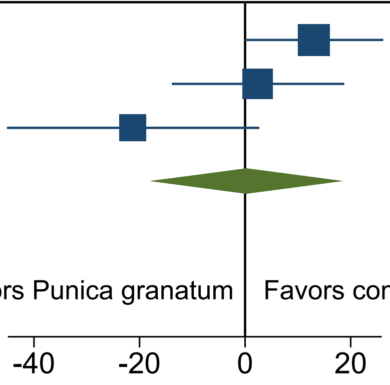

Random-effects REML model
